# Opposite Effects of Chronic Central Leptin Infusion on Activation of Insulin Signaling Pathways in Adipose Tissue and Liver Are Related to Changes in the Inflammatory Environment

**DOI:** 10.3390/biom11111734

**Published:** 2021-11-21

**Authors:** Vicente Barrios, Ana Campillo-Calatayud, Santiago Guerra-Cantera, Sandra Canelles, Álvaro Martín-Rivada, Laura M. Frago, Julie A. Chowen, Jesús Argente

**Affiliations:** 1 Department of Endocrinology, Hospital Infantil Universitario Niño Jesús, Instituto de Investigación La Princesa, E-28009 Madrid, Spain; acampilloc@salud.madrid.org (A.C.-C.); santiago.guerra@estudiante.uam.es (S.G.-C.); sandra.canelles@salud.madrid.org (S.C.); amrivada@salud.madrid.org (Á.M.-R.); laura.frago@uam.es (L.M.F.); julieann.chowen@salud.madrid.org (J.A.C.); 2 Centro de Investigación Biomédica en Red de Fisiopatología de la Obesidad y Nutrición (CIBEROBN), Instituto de Salud Carlos III, E-28009 Madrid, Spain; 3 Department of Pediatrics, Faculty of Medicine, Universidad Autónoma de Madrid, E-28029 Madrid, Spain; 4 IMDEA Food Institute, CEI UAM + CSIC, E-28049 Madrid, Spain

**Keywords:** adipose tissue, cytokines, inflammation, insulin signaling, leptin, liver

## Abstract

Leptin modulates insulin signaling and this involves the Akt pathway, which is influenced by changes in the inflammatory environment and with leptin regulating cytokine synthesis. We evaluated the association between activation of the insulin-signaling pathway and alterations in pro- and anti-inflammatory cytokine levels in inguinal fat and liver of chronic central leptin infused (L), pair-fed (PF), and control rats. Signal transducer and activator of transcription 3 (STAT3) phosphorylation was increased in inguinal fat and reduced in liver of L rats. Phosphorylation of c-Jun N-terminal kinase (JNK) and nuclear factor kappa B (NFkB) was increased in inguinal fat of L rats, together with a pro-inflammatory cytokine profile, while in the liver activation of JNK and NFkB were reduced and an anti-inflammatory pattern was found. Phosphorylation of the insulin receptor, Akt and mechanistic target of rapamycin was decreased in inguinal fat and increased in liver of L rats. There was a direct relationship between pSTAT3 and JNK and a negative correlation of Akt with pSTAT3 and JNK in both tissues. These results indicate that the effects of chronically increased leptin on insulin-related signaling are tissue-specific and suggest that inflammation plays a relevant role in the crosstalk between leptin and insulin signaling.

## 1. Introduction

Leptin and insulin are major signal molecules involved in the regulation of body weight and energy homeostasis, acting on the hypothalamus and peripheral tissues. Their signaling pathways are linked at different levels, including insulin receptor substrate (IRS) and phosphatidylinositol 3-kinase (PI3K). Leptin exerts insulin-related metabolic actions through phosphorylation of Janus kinase 2 (JAK2) that can activate IRS and subsequently PI3K [1]. The activation of this pathway triggers Akt and mechanistic target of rapamycin (mTOR), which integrates intracellular signaling and extracellular signals, such as nutrients, acting as a key regulator of cellular metabolism [2].

Although the role of central signaling crosstalk between these hormones on the maintenance of body weight and energy homeostasis is better characterized, the regulation by leptin of insulin’s actions and sensitivity also plays a key role in peripheral tissues, contributing to the regulation of energy homeostasis [3]. Leptin normally improves systemic insulin sensitivity and body glucose utilization, but this adipokine has differential effects in modulating glucose utilization depending on the tissue. For example, it augments glucose usage in brown adipose tissue (BAT) and skeletal muscle and insulin sensitivity in liver, but suppresses glucose use in white adipose tissue (WAT) [4]. These tissue-specific effects, together with oxygen usage contribute to the decrease in whole body adiposity by increasing energy consumption in BAT and muscle while reducing energy storage in WAT.

Inflammatory cytokines can impair insulin signaling in some peripheral tissue, with stress stimuli, such as increased interleukins, exacerbating the progressive development of hyperglycemia and the activation of c-jun N-terminal kinase activation (JNK) and nuclear factor kappa B (NFκB), factors involved in the reduction of insulin sensitivity [5]. In this regard, leptin activates signal transducer and activator of transcription 3 (STAT3) which can increase p38 mitogen-activated protein kinase (p38-MAPK), and in turn activating JNK [6]. This pathway augments the synthesis of proinflammatory cytokines directly or through potentiating STAT3 activation that may also enhance cytokine synthesis. These events inhibit insulin signaling at several levels, including IRS and PI3K [7,8]. The increase in the inflammatory response caused by leptin promotes the release of macrophage chemoattractant proteins, causing insulin resistance and inducing an increase in cytokines, aggravating this situation. Moreover, insulin resistance increases the synthesis of inflammatory cytokines in adipocytes, exacerbating this state [9].

Here we have analyzed the effect of chronic exposure to increased leptin levels on insulin-related signaling in inguinal fat and liver of Wistar male rats and the relationship with the inflammatory environment and leptin signaling. As it is important to distinguish between the direct effects of leptin from those due to decreased food intake that can modify insulin sensitivity and cytokine synthesis [10], a group of pair-fed rats was included. Variations in the activation of inflammatory targets and tissue cytokine pattern and their possible association with changes in insulin signaling were examined. Finally, the potential contribution of inguinal fat and liver to the circulating cytokine levels was also assessed.

## 2. Materials and Methods

### 2.1. Materials

All reagents were from Merck (Darmstadt, Germany) unless otherwise stated. Antibodies against actin were from Thermo Fisher Scientific (Waltham, MA, USA), forkhead box protein O1 (FOXO1) from Cell Signaling Technology (Danvers, MA, USA) and phosphorylated (p) phosphatase and tensin homolog on chromosome 10 (PTEN) from Santa Cruz Biotechnology (Santa Cruz, CA, USA). The Immun-Star Western C kit was from Bio-Rad Laboratories (Hercules, CA, USA). The secondary antibodies conjugated with horseradish-peroxidase were purchased from Thermo Fisher Scientific.

### 2.2. Animals

This study was designed and performed according to the European Communities Council Directive (2010/63/UE) and the Spanish Royal Decree 53/2013 concerning the protection of experimental animals. It was approved by the Ethical Committee of Animal Experimentation of the Universidad de Alcalá (PROEX018/16, 14 June 2016). The number of animals used in this study was reduced to the minimum required. A total of fifteen male Wistar rats (250 ± 10 g) purchased from Harlan Laboratories (Barcelona, Spain) were housed individually under controlled dark-light cycles (12 h/12 h), temperature (22 °C) and humidity (50%) and had access to water ad libitum. Animals were anesthetized using 4 mg of ketamine/100 g of body weight (bw) and 0.5 mg of xylazine/100 g bw throughout surgical procedures.

### 2.3. Experimental Design

After a fasting period of 12 h, rats were anesthetized and placed in a stereotaxic apparatus and treated, as described [11]. On the day of surgery, a cannula attached to an osmotic minipump (Alzet, Durect Corp., Cupertino, CA, USA) containing either saline or leptin was implanted into the right cerebral ventricle (−0.3 mm anteroposterior, 1.1 mm lateral from Bregma). Experimental animals were treated icv for 14 days with either saline with 1% BSA or leptin dissolved in saline with BSA (12 μg/day). We incorporated a pair-fed group that received the same amount of chow by the leptin-treated group the day before. This resulted in three groups: chronic saline with 1% BSA (control, C), pair-fed rats with chronic saline with 1% BSA (PF) and chronic leptin (L) with five animals per group. Animals were euthanized by decapitation at 8.00 h and the inguinal fat and whole liver, without removal of the lipids, were dissected, weighed and prepared for biochemical determinations. Peripheral blood was collected and centrifuged at 1800× *g* for 10 min at 4 °C and serum was aliquoted and frozen at −80 °C until processed.

### 2.4. Tissue Homogenization and Protein Quantification

For detection of pThr308Akt, Akt, FOXO1, interleukin (IL)-1β, (IL)-4, IL-13, IL-17A, phosphorylated (p) insulin receptor (p-IR), pSer636-IRS1, IRS1, pThr183/Tyr185-JNK, JNK, pThr180/Tyr182-p38MAPK, p38MAPK, monocyte chemoattractant protein (MCP)-1, pSer2448-mTOR, mTOR, pSer536-nuclear factor kappa B (pSer536-NFkB), NFkB, pSer380/Thr382/383-PTEN, pSer727 signal transducer and activator of transcription 3 (pSer727STAT3) and STAT3, thirty mg of inguinal fat and liver were homogenized on ice in 400 μL of lysis buffer (Merck). The lysates were frozen during 16 h at −80 °C. Later, samples were centrifuged at 12,000× *g* for 5 min at 4 °C and the supernatants kept at −80 °C until assayed [12]. Protein levels were measured by the Bradford procedure (Bio-Rad Laboratories).

### 2.5. Western Blotting

Western blotting was performed as previously described [13]. Twenty µg of protein were resolved on 10% sodium dodecyl sulphate-denaturing polyacrylamide gels, transferred to polyvinylidene difluoride membranes that were then incubated with antibodies against FOXO1 and pSer380Thr382-383PTEN. Peroxidase activity was detected by using an ECL system (Bio-Rad Laboratories) and chemiluminescent signal quantified with ImageQuant Las 4000 Software (GE Healthcare Life Sciences, Barcelona, Spain). Gel loading variability for these proteins were normalized with actin.

### 2.6. Phosphorylation of Insulin Receptor

The solid phase sandwich ELISA was from Assay Solution (Woburn, MA, USA) and detects phosphorylated insulin receptor β protein. Briefly, after incubation with the tissue lysates, insulin receptor β protein was bound by the coated monoclonal antibody. Following washing, a detection antibody coupled to biotin was incubated. A streptavidin-HRP complex was added and after extensive washing, tetramethylbenzidine was added to develop color. The absorbance is proportional to the quantity of insulin receptor phosphorylated.

### 2.7. Multiplexed Bead Immunoassays

Phosphorylated and total levels of Akt, IRS1, JNK, mTOR, NFκB, p38MAPK and STAT3 in inguinal fat and liver, as well as serum and tissue levels of IL-1β, IL-4, IL-13, IL-17A and MCP-1, were measured by multiplexed bead immunoassays (Bio-Rad Laboratories and Merck), as stated [14]. Concisely, antibodies coupled to magnetic beads and lysates were incubated overnight at 4 °C. Subsequently, after washing with a magnetic separation block (Merck), the corresponding antibodies coupled to biotin were added. After incubation for 30 min at room temperature, a streptavidin-phycoerythrin complex was added and incubated during the same-time period. At least 50 beads per analyte were examined in the Bio-Plex suspension array system 200 (Bio-Rad Laboratories). Raw data (median fluorescence intensity, MFI) were evaluated with the Bio-Plex Manager Software 4.1 (Bio-Rad Laboratories).

### 2.8. Statistical Analysis

Statistical analyses were carried out using Statview (Statview 5.01, SAS Institute, Cary, NC, USA) software. To detect differences in the parameters analyzed one-way ANOVAs were performed and then Bonferroni post hoc tests were employed. Data are represented as mean ± standard error of the mean (SEM). Linear regression analysis was employed to determine the relationships between specific parameters. In all analyses *p* < 0.05 was considered significant. Graphs were made using the software GraphPad Prism 8 (GraphPad Software, San Diego, CA, USA).

## 3. Results

### 3.1. General Characteristics of the Experimental Groups

We have previously reported that food intake and body weight gain were reduced in the PF and L groups. In addition, we also previously demonstrated that serum leptin levels were increased in rats treated centrally with leptin [15] and that serum insulin and IL-6 concentrations did not change after leptin infusion [16]. Here, we show that the percentage of inguinal fat mass was reduced in L rats and no differences in the percentage of liver weight were found among the experimental groups (Table 1).

Serum IL-1β and IL-4 concentrations were increased in L rats compared to the C and PF groups. Serum IL-17 and MCP-1 levels remained unchanged, and IL-13 was augmented in PF and L, being higher in the L group with respect to PF rats (Table 1).

### 3.2. Leptin Modifies the Activation of Inflammatory Targets and Cytokine Levels in Inguinal Fat and Liver in an Opposed Manner

**Figure 1:**
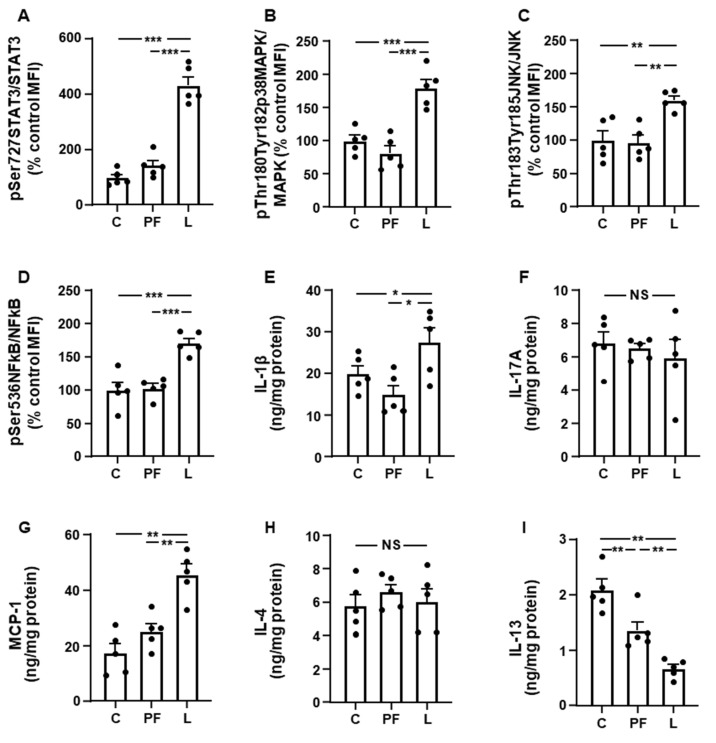
Effect of chronic central leptin infusion on inflammatory markers in inguinal fat. Relative protein levels of (**A**) signal transducer and activator of transcription 3 (STAT3) phosphorylated (p) at Ser727 (pSer727STAT3), (**B**) phosphorylated p38 mitogen-activated protein kinase (p38MAPK) at Thr180 and Tyr182 (pThr180Tyr182p38MAPK), (**C**) c-Jun N-terminal kinase (JNK) phosphorylated at Thr183 and Tyr185 (pThr183Tyr185JNK), and (**D**) nuclear factor kappa B (NFkB) phosphorylated at Ser536 (pSer536NFkB), and (**E**) interleukin (IL)1β, (**F**) IL-17A, (**G**) monocyte chemoattractant protein-1 (MCP-1), (**H**) IL-4 and (**I**) IL-13 content in the inguinal fat of control rats (**C**), pair-fed rats (PF) and rats receiving a chronic intracerebroventricular leptin infusion (L). Data are presented as means ± SEM. MFI, median fluorescence intensity; NS, non-significant. * *p* < 0.05, ** *p* < 0.01, *** *p* < 0.001.

We first determined the phosphorylation of STAT3 at Ser727 in inguinal fat and found an augment in L compared to C and PF rats (Figure 1A). Phosphorylation of p38MAPK, JNK and NFκB was increased in L rats compared to the C and PF groups (Figure 1B–D, respectively). When we analyzed the pro- and anti-inflammatory cytokines in this fat depot, we observed an increase in IL-1β levels in L rats with respect to the other groups (Figure 1E), whereas IL-17A levels remained unchanged (Figure 1F). Levels of MCP were augmented in L compared to C and PF (Figure 1G). No differences in IL-4 concentrations were found (Figure 1H) and IL-13 levels were reduced in both PF and L rats, with this reduction being more pronounced in the L group (Figure 1I).

**Figure 2:**
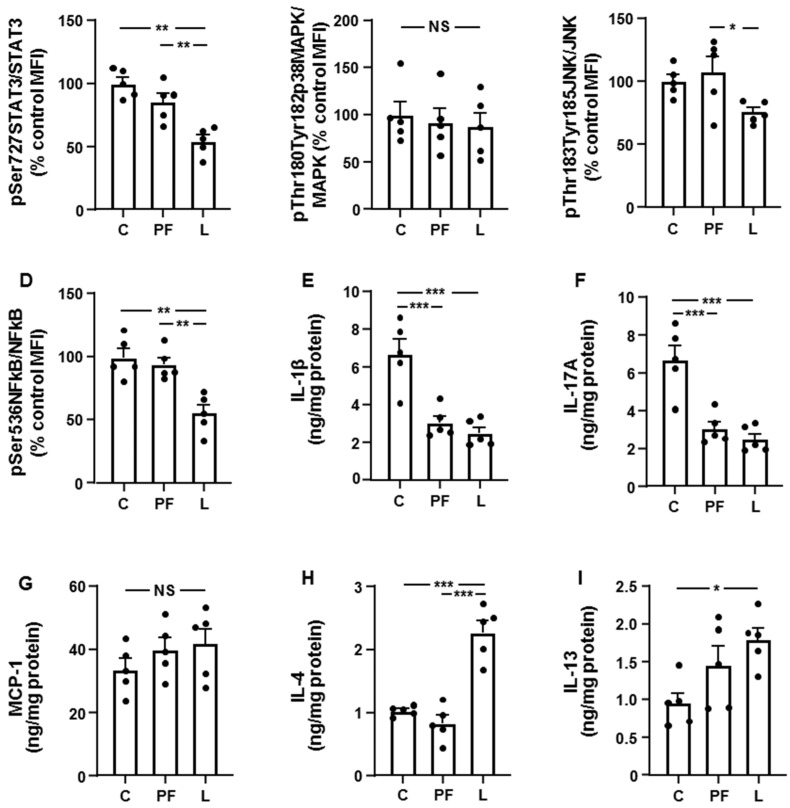
Effect of leptin infusion on inflammatory markers in the liver. Relative protein levels of (**A**) signal transducer and activator of transcription 3 (STAT3) phosphorylated (p) at Ser727 (pSer727STAT3), (**B**) phosphorylated p38 mitogen-activated protein kinase (p38MAPK) at Thr180 and Tyr182 (pThr180Tyr182p38MAPK), (**C**) c-Jun N-terminal kinase (JNK) phosphorylated at Thr183 and Tyr185 (pThr183Tyr185JNK), and (**D**) nuclear factor kappa B (NFkB) phosphorylated at Ser536 (pSer536NFkB), and (**E**) interleukin (IL)1β, (**F**) IL-17A, (**G**) monocyte chemoattractant protein-1 (MCP-1), (**H**) IL-4 and (**I**) IL-13 content in the liver of control rats (**C**), pair-fed rats (PF) and rats receiving a chronic intracerebroventricular leptin infusion (L). Data are presented as means ± SEM. MFI, median fluorescence intensity; NS, non-significant. * *p* < 0.05, ** *p* < 0.01, *** *p* < 0.001.

We next analyzed the same parameters in the liver. STAT3 phosphorylation was diminished in L rats compared to the other groups (Figure 2A) and no change in p38MAPK activation was found (Figure 2B). Phosphorylation of JNK was reduced in the L group compared to PF rats (Figure 2C) and NFκB activation was decreased in L with respect to C and PF animals (Figure 2D). Hepatic levels of IL-1β and IL-17A were reduced in both PF and L groups (Figure 2E,F, respectively). MCP-1 protein levels were not different between the three groups (Figure 2G) and IL-4 concentrations were augmented in the L group compared to C and PF rats (Figure 2H). Finally, hepatic IL-13 concentrations were increased in L compared to C rats (Figure 2I).

### 3.3. Inguinal IRS1/PI3K Signaling Is Reduced in Leptin-Treated Rats

**Figure 3:**
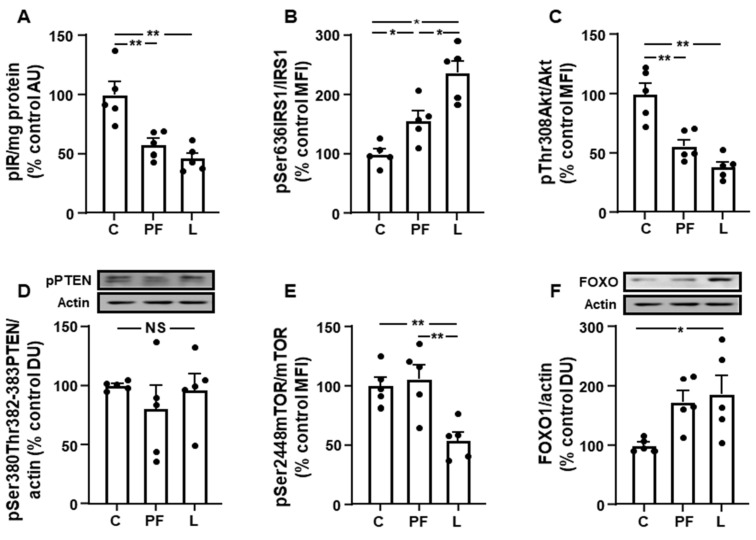
Leptin infusion reduces insulin-related signaling in the subcutaneous fat. Relative protein levels of (**A**) phosphorylated (p) insulin receptor (pIR), (**B**) insulin receptor substrate 1 (IRS1) phosphorylated at Ser636 (pSer636IRS1), (**C**) Akt phosphorylated at Thr308 (pThr308Akt), (**D**) phosphatase and tensin homologue deleted on chromosome 10 (PTEN) phosphorylated at Ser380 and Thr382-383 (pSer380Thr382-383PTEN), (**E**) mammalian target of rapamycin (mTOR) phosphorylated at Ser2448 (pSer2448mTOR) and (**F**) forkhead box O1 (FOXO1) in the subcutaneous adipose tissue of control rats (**C**), pair-fed rats (PF) and rats receiving a chronic intracerebroventricular leptin infusion (L). Data are presented as means ± SEM. AU, absorbance units; DU, densitometry units; MFI, median fluorescence intensity; NS, non-significant. * *p* < 0.05, ** *p* < 0.01.

Phosphorylation of the insulin receptor was reduced in inguinal fat of the PF and L groups (Figure 3A). Phosphorylation of IRS1 on serine residues attenuates the PI3K pathway [17] and we found an increase in PF and L, with this being greater in the L group (Figure 3B), whereas Akt activation was diminished in both PF and L animals (Figure 3C). Phosphorylation of PTEN was unchanged (Figure 3D) and mTOR phosphorylation in Ser2448 was decreased in L with respect to C and PF rats (Figure 3E). Relative FOXO1 protein concentrations were increased in the L group compared to C rats (Figure 3F).

### 3.4. Chronic Leptin Exposure Increases Hepatic IRS1/PI3K Signaling

**Figure 4:**
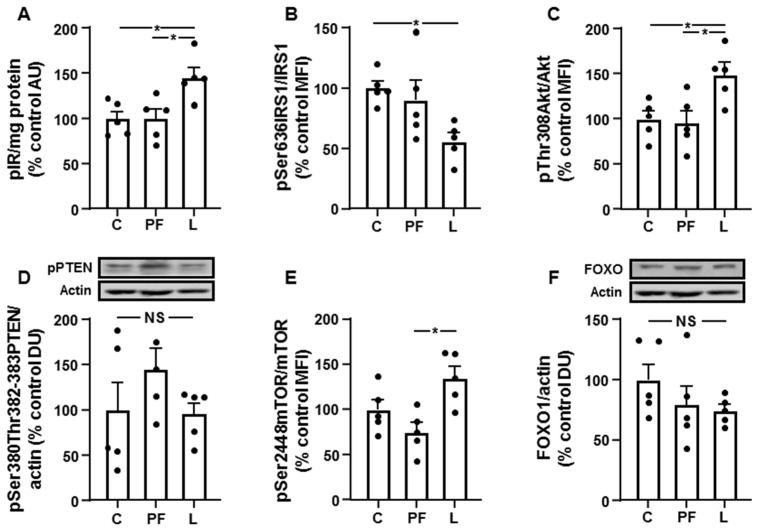
Leptin increases hepatic Akt signaling. Relative protein levels of (**A**) phosphorylated (p) insulin receptor (pIR), (**B**) insulin receptor substrate 1 (IRS1) phosphorylated at Ser636 (pSer636IRS1), (**C**) Akt phosphorylated at Thr308 (pThr308Akt), (**D**) phosphatase and tensin homologue deleted on chromosome 10 (PTEN) phosphorylated at Ser380 and Thr382-383 (pSer380Thr382-383PTEN), (**E**) mammalian target of rapamycin (mTOR) phosphorylated at Ser2448 (pSer2448mTOR) and (**F**) forkhead box O1 (FOXO1) in the liver of control rats (**C**), pair-fed rats (PF) and rats receiving a chronic intracerebroventricular leptin infusion (L). Data are presented as means ± SEM. AU, absorbance units; DU, densitometry units; MFI, median fluorescence intensity; NS, non-significant. * *p* < 0.05.

Activation of insulin receptor was augmented in the L group compared to C and PF rats (Figure 4A), and phosphorylation of IRS1 at Ser636 reduced in L rats (Figure 4B). Akt activation, measured as phosphorylation at Thr307, was augmented in the L group with respect to C and PF rats (Figure 4C) and PTEN phosphorylation was similar in all experimental groups (Figure 4D). Phosphorylation of mTOR was increased in L rats compared to the PF group (Figure 4E). Relative FOXO1 protein levels did not show significant differences among the three experimental groups (Figure 4F).

### 3.5. Changes in Akt Activation Are Inversely Related to STAT3 and JNK Phosphorylation

**Figure 5:**
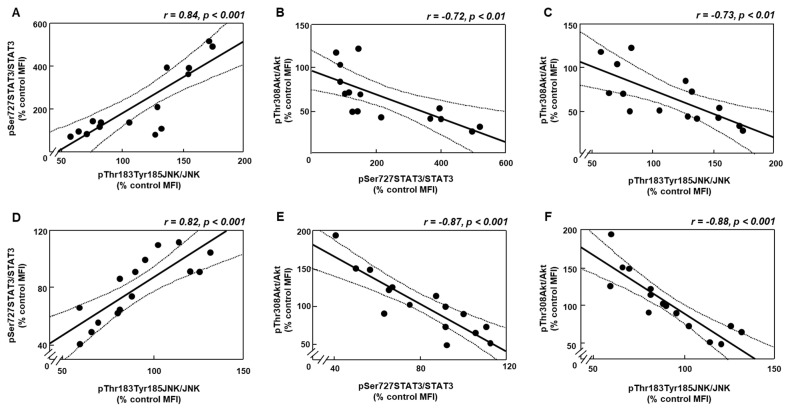
Correlations between leptin-, inflammatory- and insulin-related targets. (**A**) Linear correlation between relative protein levels of signal transducer and activator of transcription 3 (STAT3) phosphorylated (p) at Ser727 (pSer727STAT3) and c-Jun N-terminal kinase (JNK) phosphorylated at Thr183 and Tyr185 (pThr183Tyr185JNK) in the inguinal fat. (**B**) Linear correlation between relative protein levels of Akt phosphorylated at Thr308 (pThr308Akt) and pSer727STAT3 in the inguinal fat. (**C**) Linear correlation between relative protein levels of pThr308Akt and pThr183Tyr185JNK in the inguinal fat. (**D**) Linear correlation between relative protein levels of pSer727STAT3 and pThr183Tyr185JNK in the liver. (**E**) Linear correlation between relative protein levels of pThr308Akt and pSer727STAT3 in the liver. (**F**) Linear correlation between relative protein levels of pThr308Akt and pThr183Tyr185JNK in the liver. MFI, median fluorescent intensity. The 95% confidence interval is indicated by hatched curves. Correlation coefficients (r) and *p* values are represented for each analysis.

As leptin is involved in the regulation of several inflammatory mediators that may attenuate insulin-related signaling [18,19], we analyzed the possible relationship among STAT3, JNK and Akt phosphorylation in inguinal fat and liver. Linear regression analyses showed a positive correlation of pSTAT3 with pJNK in fat and liver (Figure 5A,D, respectively) and inverse of pAkt with pSTAT3 (Figure 5B,E, respectively) and JNK (Figure 5C,F, respectively).

## 4. Discussion

Leptin regulates substrate utilization in a tissues-specific manner, suppressing glucose use in WAT and promoting it in other tissues such as liver, with these changes paralleling insulin sensitivity in these tissues [20]. This study demonstrates that chronic central leptin infusion caused opposite responses in insulin signaling in inguinal fat and liver that appear to be related to the local inflammatory environment. Moreover, a direct effect of leptin most likely occurs, since most of the analyzed parameters did not differ between pair-fed rats and controls. Finally, our results suggest that both tissues may contribute to the changes in circulating cytokine levels observed in leptin-treated rats.

One of the most important findings shown here is the activation of several inflammatory targets in inguinal fat. This could be related to the susceptibility of adipose tissue to changes in circulating leptin levels. Indeed, we previously reported hyperleptinemia in this experimental model [13], a situation that provokes immunometabolic changes in different cell types, including adipose tissue resident macrophages. We found an increase in phosphorylation of p38MAPK, JNK and NFκB together with an augment in the tissue concentrations of proinflammatory cytokines.

Leptin activates immune cells through the MAPK pathway that activates NFκB, which participates in the intracellular signal transduction and production of inflammatory cytokines and chemokines [21]. Under normal conditions adipose tissue macrophages present an anti-inflammatory phenotype; however, after leptin administration, a polarization to a proinflammatory phenotype of these cells takes place [22], which could at least partially explain, the cytokine pattern observed in inguinal fat in response to leptin. Once activated macrophages, in combination with adipocytes and other cell types, could promote macrophage recruitment and synthesis of proinflammatory cytokines [23].

Central leptin infusion did not activate inflammatory targets in the liver, but reduced phosphorylation of NFκB. Leptin administration was reported to promote PI3K-dependent increase in hepatic fatty acid oxidation and decrease in triglyceride levels. These actions on liver-resident immune cells are required for these metabolic effects, as myeloid cell-specific deletion of the leptin receptor or depletion of liver Kupffer cells reduced these metabolic effects, whereas deletion of leptin receptors in hepatocytes did not affect hepatic metabolism [24]. In addition, the liver is less vulnerable than adipose tissue to the increase in inflammatory macrophages after the development of diet-induced obesity [25], which could explain the absence of an inflammatory profile here.

The inverse pattern in STAT3 activation in these tissues after leptin administration is a relevant finding of this study. STAT3 phosphorylated on Ser727 coordinates innate immune mitochondrial reprogramming and inflammatory cell metabolism. The increase in STAT3 phosphorylation in inguinal fat may be due to serine 727 residue being embedded in a consensus cyclin-dependent kinase and MAPK motif and is phosphorylated by p38MAPK and JNK, among other kinases [26]. In addition, the rise in STAT3 activation can trigger inflammation in fat as this phosphorylation is required for IL-1β synthesis [27].

The reduction in hepatic STAT3 phosphorylation could be related to an anti-inflammatory cytokine pattern since its activation is key in chronic low-grade inflammation [28]. STAT3 is an important transcription factor in the differentiation of T helper 17 (Th17) cells, involved in chronic inflammation. The differentiation of Th17 cells is regulated by multiple cytokines, including IL-4 [29]. These cells secrete interleukins, stimulating the production of pro-inflammatory molecules implicated in the pathogenesis of metabolic diseases [30]. Hence, the reduction in hepatic levels of IL-17, together with the increase in IL-4 could be involved in the anti-inflammatory profile found in the liver of leptin-treated rats. Although no changes in the activation of p38MAPK or JNK were found in the liver, which could explain the decrease in STAT3 activation, the reduction in NFκB phosphorylation could be involved since NFkB-dependent activation of STAT3 has been reported [31]. Furthermore, chronic leptin infusion reduces fat mass and abolishes hepatic STAT-3 phosphorylation in response to acute leptin injection [32].

The reduction in fat depots observed in this study is probably due to leptin’s sup-pression of glucose metabolism [4] and insulin sensitivity. In adipose tissue, insulin pro-motes glucose transport and lipogenesis and inhibits lipolysis, reducing substrate supply for gluconeogenesis in the liver [33], whereas a decrease in insulin sensitivity could alter these pathways, causing metabolic disturbances and inflammation. Although most of the effects of leptin infusion differed from those in pair-feed animals, and thus are most likely not due to the reduction in food intake, pair-fed rats had reduced insulin signaling in inguinal fat, although this reduction was less than that in leptin-treated rats. Acute fasting can improve insulin signaling, but reduction of food intake during long periods can augment insulin resistance [34]. This effect was reported to be due to a diminution in the phosphorylation of insulin receptor and Akt, as found here.

Inflammation participates in the regulation of feeding and body gain, but it can also provoke insulin resistance independently of modifications in body weight. We found a reduction in the phosphorylation of the insulin receptor, IRS1 in serine 636 and Akt, together with an increase in p38MAPK and JNK activation in fat of leptin-treated rats leading to inhibition of insulin’s actions in adipose cells [35]. On the other hand, phosphorylation on serine residues of IRS1 and JNK can interfere with insulin signaling [17] by favoring the degradation of IRS1 and promoting IRS1 serine phosphorylation in WAT, thus decreasing signal transduction and increasing inflammation [36]. Another mechanism is mediated by STAT3 phosphorylation at serine 727 that impairs insulin signaling, inhibiting insulin-stimulated Akt phosphorylation [37]. Here an inverse inflammatory pattern was found in the liver, and this could explain the increased activation of the PI3K/Akt pathway. Indeed, pharmacological downregulation of p38MAPK and JNK in type 2 diabetic rats was associated with the potentiation of hepatic insulin signaling [38].

The PI3K/Akt pathway also regulates cytokine expression. We found reduced mTOR phosphorylation in inguinal fat and increased in the liver of leptin-treated rats. Several studies have reported that the mTOR complex 1 (mTORC1) constrains immune cell activation by upregulation of anti-inflammatory interleukins and inhibition of proinflammatory cytokines [39,40]. Our results suggest that the increase in inflammatory cytokines in inguinal fat could be due, at least in part, to increased FOXO1. When mTOR is activated, it stimulates FOXO1 phosphorylation and translocation to the cytosol and degraded, enabling genes, such as those for the proinflammatory factors IL-1β and MCP-1, among others, to be expressed [41], whereas downregulation of the Akt pathway reduces IL-4 production stabilizing FOXO1 complexes [42]. In brief, the PI3K/AKT pathway inhibits FoxO1 via phosphorylation and subsequent nuclear exclusion allowing the expression of targeted genes, whereas activation of stress-activated kinases, such as JNK and MAPK, maintains an inflammatory status and dysregulates metabolism by antagonizing insulin signaling transduction [43,44].

Our study has several limitations. It demonstrates that leptin modifies activation of insulin signaling associated to modifications in inflammatory targets, however, it remains to be elucidated to what degree the inflammatory cascade and leptin signaling contribute to the differential activation of insulin targets in fat and liver after leptin infusion. Moreover, measuring the tissue levels of a cytokine does not necessarily reflect their contribution to circulating levels. One must also take into consideration that the leptin dose and duration of treatment could also affect the outcomes described here. This dose and time period were chosen as they were previously reported to provoke changes in peripheral insulin sensitivity [45]; however, we cannot discard the possibility that the dose-response of different tissues to this leptin treatment, including that of liver and inguinal fat, differs. Lower leptin doses than the used here have been shown to improve insulin sensitivity, while decreasing the in vivo insulin action in WAT, including decreasing insulin receptor phosphorylation and activation of the downstream insulin pathway [46]. The time at which these parameters are measured will also clearly affect the outcome as leptin treatment produces changes in weight gain and effects on other tissues [47,48] that could in turn affect those tissues analyzed here. However, this does not negate the observation that liver and inguinal adipose tissue have differential responses to central leptin treatment at this timepoint. Finally, we did not examine cytokine expression and protein levels in other tissues, such as skeletal muscle, that can also affect the circulating cytokine profile.

## 5. Conclusions

**Figure 6:**
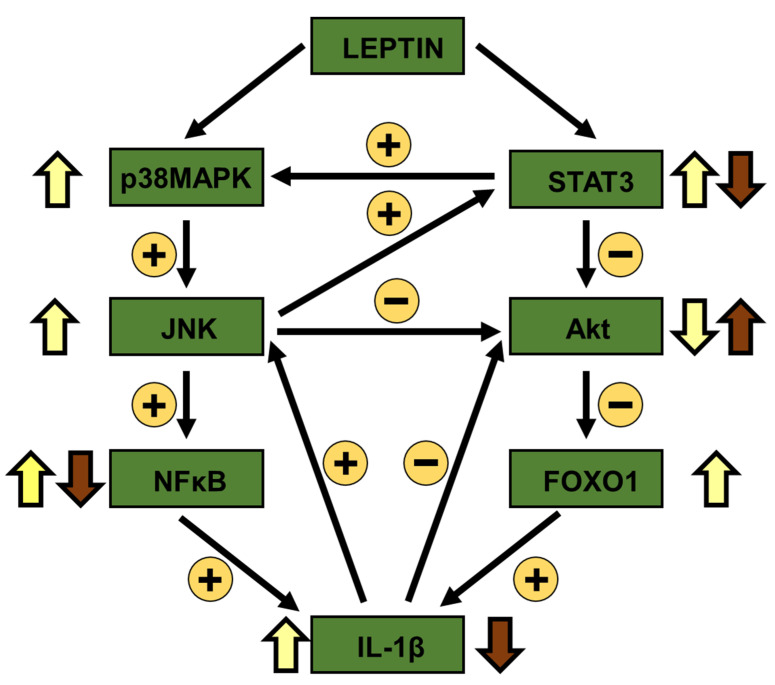
Proposed mechanism of action. Treatment with leptin increases inflammatory pathways in inguinal fat, stimulating proinflammatory cytokines, as IL-1β. In addition, the augment in the activation of STAT3 in this fat depot may increase phosphorylation of p38MAPK and reduce Akt-related signaling, increasing FOXO1 levels that also promote an inflammatory environment. Proinflammatory cytokines activate inflammatory targets and inhibit Akt-related signaling. On the other hand, the reduction in leptin-related signaling in the liver may favor the increase in Akt activation that inhibits FOXO1. This fact could promote an anti-inflammatory environment in the liver of leptin-treated rats. Activation (or increase) and inhibition (or reduction) in inguinal fat (yellow arrows) or liver (brown arrows) is indicated. Akt, protein kinase B; FOXO1, forkhead box O1; JNK, c-Jun N-terminal kinase; IL, interleukin; NFκB, nuclear factor kappa B; p38MAPK, p38 mitogen-activated protein kinase; STAT3, signal transducer and activator of transcription 3.

As summarized in Figure 6, our results suggest that an increase in leptin levels is implicated in changes in the activation of several inflammatory mediators, as well as in the cytokine profile in inguinal fat and liver through modifications in leptin-related signaling. These changes in the inflammatory environment could vary insulin-related signaling in both locations. Differential changes in STAT3 phosphorylation could indicate that this factor is a molecular target that controls inflammatory pathways and local cytokine levels, inversely modifying the activation of insulin pathway in inguinal fat and liver. These findings could indicate that the previously reported tissue-specific effects of leptin on glucose utilization may be governed by variations in inflammation and subsequently in insulin sensitivity and suggest that pharmacological intervention of leptin signaling might be a strategy for controlling pathophysiological situations where insulin sensitivity is altered.

## Figures and Tables

**Table 1:** Weight of subcutaneous fat and liver and circulating levels of cytokines.

Parameter	Control	Pair-Fed	Leptin
Inguinal fat weight (%)	1.02 ± 0.08	0.94 ± 0.09	0.64 ± 0.08 ** ^##^
Liver weight (%)	3.62 ± 0.23	3.82 ± 0.13	3.99 ± 0.10
Serum IL-1β (pg/mL)	25.56 ± 2.63	17.63 ± 2.40	39.32 ± 4.77 * ^#^
Serum IL-17A (pg/mL)	8.95 ± 1.55	7.51 ± 1.02	6.40 ± 0.78
Serum MCP-1 (pg/mL)	36.96 ± 3.82	31.48 ± 2.87	36.86 ± 3.67
Serum IL-4 (pg/mL)	3.25 ± 0.31	2.93 ± 0.60	11.09 ± 1.00 *** ^###^
Serum IL-13 (pg/mL)	1.95 ± 0.18	3.13 ± 0.30 *	5.65 ± 0.62 * ^#^

Values are means ± SEM of five animals. IL, interleukin; MCP-1; monocyte chemoattractant protein-1. * *p* < 0.05, ** *p* < 0.01, *** *p* < 0.001 vs. C; ^#^ *p* < 0.05, ^##^ *p* < 0.01, ^###^ *p* < 0.001 vs. PF. The percentage of inguinal fat and liver weight was calculated in relationship to the total rat body weight.

## Data Availability

All relevant data are included within the manuscript.
